# Glutamate receptors within the lateral periaqueductal gray (lPAG): Effects on blood pressure and heart rate in basic and hypotensive hemorrhagic rats

**DOI:** 10.22038/ijbms.2025.80366.17399

**Published:** 2025

**Authors:** Vida Alikhani, Reza Mohebbati, Mohammad Naser Shafei

**Affiliations:** 1 Department of Physiology, Faculty of Medicine, Gonabad University of Medical Sciences, Gonabad, Iran; 2 Applied Biomedical Research Center, Mashhad University of Medical Sciences, Mashhad, Iran; 3 Department of Physiology, Faculty of Medicine, Mashhad University of Medical Sciences, Mashhad, Iran; 4 Division of Neurocognitive Sciences, Psychiatry and Behavioral Sciences Research Center, Mashhad University of Medical Sciences, Mashhad, Iran

**Keywords:** Cardiovascular system, Glutamate, Hemorrhage, Lateral periaqueductal gray, Non-NMDA receptor

## Abstract

**Objective(s):**

The lateral periaqueductal gray (lPAG) is involved in regulating cardiovascular activity. As glutamate is present in this region, the effect of glutamatergic receptors on cardiovascular functions in basic and hypotensive hemorrhagic (Hem) rats was investigated.

**Materials and Methods:**

In both basic and hemorrhagic animals, saline, L-glutamate (L-Glu), and ionotropic receptor antagonists (MK-801 and GYK as NMDA and non-NMDA receptor antagonists, respectively) with and without L-Glu microinjected into the lPAG. The heart rate (HR), mean arterial pressure (MAP), and systolic blood pressure (SBP) were captured, and those changes (Δ) were calculated and analyzed.

**Results:**

The results showed that in basic conditions, microinjection of MK-801 and GYK alone did not significantly affect cardiovascular parameters, but L-Glu significantly increased all parameters (*P*<0.001). However, co-injection of GYK and L-Glu significantly attenuated cardiovascular responses induced by L-Glu (*P*<0.01), whereas MK-801 did not have effects. In Hem hypotensive condition, injection of MK-801 and GYK alone could not significantly change the cardiovascular responses, while L-Glu alone significantly increased these responses (*P*<0.001). In this condition, Co-injection of L-Glu with GYK attenuates the pressor effect of L-Glu, but MK-801 did not affect it.

**Conclusion:**

Findings suggest an excitatory cardiovascular role of the glutamatergic system of lPAG in normotensive conditions mediated by a non-NMDA receptor. However, in hem hypotensive conditions, the endogenous glutamatergic system of lPAG does not affect hem hypotension, while exogenous glutamate via a non-NMDA receptor could improve cardiovascular responses in this condition.

## Introduction

Numerous brain areas, most of which are located in the brainstem, affect central regulation of cardiovascular activity. The major neuroregulatory centers of the cardiovascular system include the Rostral ventrolateral medulla (RVLM), which regulates blood pressure and HR by acting on sympathetic pre-ganglion neurons (1), the nucleus tractus solitarius (NTS), hypothalamic nuclei, and different parts of the periaqueductal gray matter (PAG). These areas cause appropriate cardiovascular responses during conditions such as defensive reactions, pain, and hemorrhage (2). 

The PAG area is categorized into four distinct columns: dorsolateral, dorsomedial, lateral, and ventrolateral or (dlPAG), (dmPAG), (lPAG), and (vlPAG), respectively. The lPAG column has an important role in pain modulation, defensive reactions, fear responses, the respiratory system, and the cardiovascular system (3). It is also responsible for increasing blood pressure, heart rate, respiratory rate, and constricting mesenteric vessels during stress (4, 5). The lPAG is also involved in regulating cardiovascular activity through tachycardia and hypertension during defense responses (6). 

Studies have shown that the lateral PAG sends projections to brain regions that regulate cardiovascular functions, including RVLM and NTS (7, 8). For example, it has been reported that the lPAG region contains neurons that influence cardiovascular neurons in the RVLM (9), and the lPAG projections to NK1R-ir neurons in the RVLM are involved in cardiovascular responses to stress or other physiological stimuli (8). Additionally, it has been documented that the lPAG has a sympathetic effect mediated by communication with the spinal cord and hypothalamus (10). Stimulation of different regions of the PAG leads to different cardiovascular responses depending on the injection site. Thus, different neuronal groups in the PAG mediate cardiovascular effects (11).

The dlPAG contains neurotransmitters such as glutamate, GABA, Ach, and opioids (12, 13). Most projecting neurons from the lPAG to the RVLM use glutamate as their neurotransmitter (7, 8). The non-NMDA, NMDA receptors, and the VGLUT2 transporter of glutamate are expressed in the lPAG (7, 8). Furthermore, glutamate receptor antagonists into the l/dl PAG prevents the cardiovascular effects of steress. Therefore, the neuronal excitation by stress including increased of heart rate and mean arterial pressure mediated by glutamate receptors (14). In a recent study, we showed that the cholinergic system of this area is involved in cardiovascular regulation in normotensive and hypotensive rats (15). 

Although the presence of glutamate and the ionotropic receptors, including non-NMDA and NMDA, in the lPAG has been revealed, the role of the glutamatergic system of the lPAG in regulating cardiac and vascular activities remains unclear. Therefore, the current research aims to explore the effect of the glutamatergic system of the lPAG on cardiovascular function during basic and hemorrhagic hypotension conditions.

## Materials and Methods

### Subjects

Seventy-two male Wistar rats weighing approximately 250 ± 20g were provided from the animal house at the Faculty of Medicine, Mashhad University of Medical Sciences (MUMS). They were held in an environment with a 12-hour light/dark cycle and unrestricted access to food and water. The study protocol received approval from the Ethics Committee at MUMS (ethical code: IR.MUMS.MEDICAL.REC.1400.268). 

### Pharmacological agents

The substances administered during the experiment included urethane, glutamate (L-Glu), a glutamatergic receptor agonist, GYKI-52466 (1-(4-aminophenyl)-4-methyl-7,8-methylenedioxy-5H-2,3-benzodiazepine), a selective non-competitive antagonist for non-NMDA receptors, and MK-801, a selective NMDA receptor antagonist. Sigma Aldrich Chemical Co., USA, supplied all pharmacological agents (16, 17). 

### Grouping of animals

The rats were grouped into two main categories: (A) normotensive and (B) hypotensive hemorrhagic (Hem) groups. Each category was further subdivided into specific groups, with six rats assigned to each subgroup (n=six):

A) Normotensive groups: 1) Saline, 2) L-Glutamate (L-Glu), 3) GYK, 4) Co-injection of GYK + L-Glu, 5) MK-801, 6) Co-injection of MK-801 + L-Glu

B) Hemorrhagic (Hem) groups: In these groups, hemorrhage was induced first, followed by microinjection of the drugs: 1) Saline, 2) Glutamate (L-Glu), 3) GYK, 4) Co-injection of GYK + L-Glu, 5) MK-801, and 6) Co-injection of MK-801 + L-Glu. The doses administered for L-Glu, GYK, and MK-801 were 50, 300, and 0.5 nanomol, respectively. All substances were microinjected into the lPAG (18, 19) with a total volume of 100-150 nl for each drug (20).

### Cannula implantation and recording of cardiovascular parameters

Anesthesia was induced through an intraperitoneal urethane injection at a dosage of 1.5 grams per kilogram (20). To measure cardiovascular responses, a 22-gauge heparinized angiocatheter was placed into the femoral artery (20) and linked to a blood pressure transducer connected to a PowerLab system from ID Instrument in Australia. In the experimental groups, cardiovascular parameters were recorded for about 10 min (stabilization time). After that, to perform the study, each parameter was recorded for 5 min (before injection). Then, the drugs were injected, and the responses were recorded for 30 min (after injection). The changes in each cardiovascular parameter were calculated using Lab Chart 8 software of the Power lab device. In summary, the maximum difference (Δ, difference between pre-injection and peak changes induced by the drug) was calculated, considered the maximum change, and analyzed. A warmer was employed throughout the experiment to hold the animals’ body temperature at 37.5 °C.

### Microinjection and stereotactic surgery

Following the artery cannulation, the animal was positioned on the stereotactic frame (Stoelting, USA) with its head immobilized. The coordinates for the lPAG area were determined using a rat brain atlas (AP:5.2-8.4, L:0.2-1.5, H: 4.3-5.8) (21). Subsequently, a hole with a diameter of approximately 2 mm was then drilled into the skull, allowing for the injection of drugs into the lPAG by a micropipette with a diameter of 35-40 µm. This micropipette was attached to a syringe, and the injection was performed manually using a manual injector (Stoelting, USA) (22). 

### Hem protocol

In the Hem groups, once the cardiovascular parameters were stabilized (approximately 10 min), 15 percent of the Total Blood Volume (TBV) was gradually extracted over 10 min (from the 5^th^ to the 15^th^ minute) through the femoral artery cannula (20). TBV was calculated using the following equation:

0.06 ml per g× Body Weight+0.77 (23). Withdrawing 15% volume resulted in approximately 30 mmHg decrease in systolic blood pressure (SBP). This established appropriate circumstances for evaluating the central cardiovascular regions associated with Hem (23). After induction of Hem and steady cardiovascular parameters (about 5 min), drugs were microinjected, and changes in cardiovascular response were captured in 30 min. At the conclusion of the experiment, the animals were put to euthanasia using a lethal dose of urethane. Following this, their brains were extracted from the skulls and preserved in 10% formalin for 24 hr to ensure tissue fixation. Afterward, thin slices with a thickness of 60 microns were obtained using a vibratome. Each slide was checked by a light microscope to confirm the accurate placement of microinjections based on the rat brain atlas (24). Animals whose injection site was not according to the Paxinus atlas were excluded from the study. 

### Data evaluation 

The data are presented as mean ± standard error of the mean (SEM). The maximum alterations in cardiovascular metrics, such as mean arterial pressure (MAP), heart rate (HR), and systolic blood pressure (SBP), were documented, and their respective changes (Δ) were computed to evaluate the variations. The peak alterations were examined using a one-way ANOVA accompanied by Tukey’s *post hoc* test. A *P*-value of less than 0.05 was deemed statistically significant. 

### Mean arterial pressure calculation

MAP was calculated using either of the following formulas:

1. MAP= DP + ⅓ (SP - DP) 

2. MAP= DP + ⅓ (PP) 

Where: DP= Diastolic Pressure, SP= Systolic Pressure and PP= Pulse Pressure (SP - DP)

## Results

### Cardiovascular reactions following saline microinjection into the lPAG in normotensive rats

This section investigates the cardiovascular effects of saline microinjection, comparing measurements taken before and after the procedure. Before saline was injected, the mean arterial pressure (MAP), systolic blood pressure (SBP), and heart rate (HR) were captured at 112±11.15 mmHg, 134.9±9.4 mmHg, and 380.9±20.12 beats per minute, respectively. Post-injection, no significant alterations were observed in these parameters (MAP: 113.4 ± 12.6 mmHg, SBP: 136.3 ± 11.2 mmHg, HR: 383.3 ± 17.6 beats/min), as detailed in Table 1.

### Cardiovascular responses following GYK and MK- 801 microinjection into the lPAG in normotensive rats

**Figure 1:**
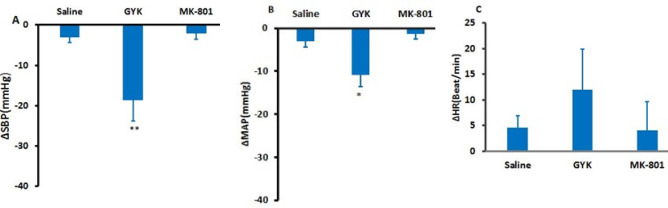
Maximal changes (∆) of systolic blood pressure (∆SBP, A), mean arterial pressure (∆MAP, B), and heart rate (∆HR, C) following saline, GYK, and MK-801 microinjection into the lPAG column in normotensive rats

Initially, saline, GYK, and MK-801 were injected into the lPAG in separate groups, and subsequently, the maximal changes in cardiovascular responses were assessed. Microinjection of saline did not significantly alter maximal changes of factors compared to pre-injection [ΔMAP (-3.22± 1.2 mmHg), ΔSBP (-4.5 ±1.35 mmHg), and ∆HR (4.6±2.3 Beat/min)]. Microinjection of MK-801 did not change ∆SBP (-2.14± 1.45 mmHg), ∆MAP (-1.45±1.2 mmHg), and ∆HR (4.45±9.7 Beat/min) vs the saline group. However, the injection of GYK significantly reduced the ∆SBP (-18.62 ± 5.2 mmHg) and ∆MAP (-10.38 ± 2.70 mmHg) compared to the saline group, with statistical significance observed (*P*<0.01 related to ∆SBP and *P*<0.05 referring to ∆MAP, as shown in Figure 1).

### Effect of microinjection of L-Glu alone and co-injected with GYK and MK-801 into the lPAG column on cardiovascular responses

**Figure 2:**
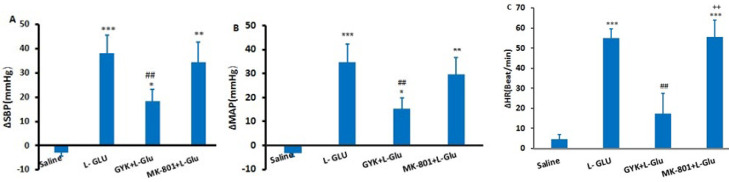
Maximum changes (∆) of systolic blood pressure (∆SBP, A), mean arterial pressure (∆MAP, B), and heart rate (∆HR, C) following saline, L-Glu, GYK+ L-Glu, and MK-801+ L-Glu microinjection in the lPAG column in normotensive rats

To better understand the cardiovascular impact of Glutamate, L-Glu was administered alone and in combination with both NMDA and non-NMDA antagonists through co-injection into the lateral periaqueductal gray (lPAG) in different experimental groups. The microinjection of saline did not produce any significant change in the maximum quantities of the measured parameters compared to pre-injection levels, which were recorded as follows: ΔMAP (-3.22 ± 1.2 mmHg), ΔSBP (-4.2 ± 1.35 mmHg), and ΔHR (4.6 ± 2.3 beats per minute). Conversely, administering L-Glu led to a substantial increase in ΔSBP (39.16 ± 7.5 mmHg), ΔMAP (36.65 ± 7.6 mmHg), and ΔHR (55 ± 4.7 beats/min; *P*<0.001) vs the saline group (Figure 2).

When GYK and L-Glu were co-injected, there was a marked reduction in ΔSBP (18.41 ± 4.8 mmHg) and ΔMAP (15.38 ± 4.5 mmHg) relative to the L-Glu (*P*<0.01). Furthermore, ΔHR was significantly lower than that obtained in the L-Glu-only group (17.32 ± 10.26 beats/min; *P*<0.01). However, co-injecting MK-801 with L-Glu into the lPAG did not lead to a significant decrease in ΔSBP (34.4 ± 8.36 mmHg) or ΔMAP (29.7 ± 7.20 mmHg) compared to the L-Glu (*P*>0.05). Additionally, the measurements were significantly more than those recorded in the saline group (*P*<0.01 - *P*<0.001).

### Cardiovascular responses following saline, GYK, and MK-801 microinjection into the lPAG in Hem hypotensive rats

**Figure 3:**
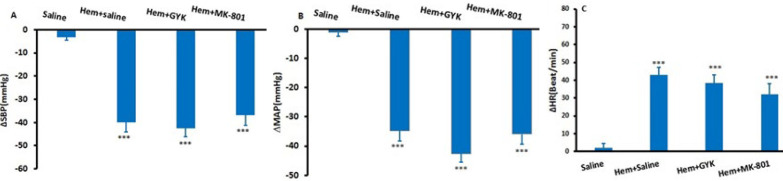
Maximum changes (∆) of systolic blood pressure (∆SBP, A), mean arterial pressure (∆MAP, B), and heart rate (∆HR, C) following hemorrhage and microinjection of saline, GYK, and MK-801 into the lPAG column in rats

The objective was to investigate the involvement of the glutamatergic system in the lPAG during hypotension induced by Hem. Five minutes following the onset of hypotension, GYK and MK-801 were each microinjected separately into the lPAG. The maximum alterations in ∆SBP, ∆MAP, and ∆HR for both the Hem group and the groups receiving GYK and MK-801 are illustrated in Figure 3. It was observed that the induction of Hem led to a significant reduction in ∆SBP (-39.9±4.2 mmHg) and ∆MAP (-34.7±3.6 mmHg) vs the saline group (*P*<0.001) while ∆HR significantly increased compared to the control group (42.85± 4.45; *P*<0.001). The changes in ∆SBP (-42.50±3.7 mmHg), ∆MAP (-37.8±2.9 mmHg), and ∆HR(38.5±4.32 Beat/min) induced by GYK (Hem +GYK)were not significant with respect to Hem+ saline group (*P*>0.05). In addition, maximal alterations of cardiovascular parameters after injection of MK-801 were not significant with respect to the Hem+ saline group (*P*>0.05, Figure 3).

### Cardiovascular responses following Glu, GYK+ L-Glu, and MK-801 +L-Glu microinjection into the lPAG in Hem hypotensive rats

**Figure 4:**
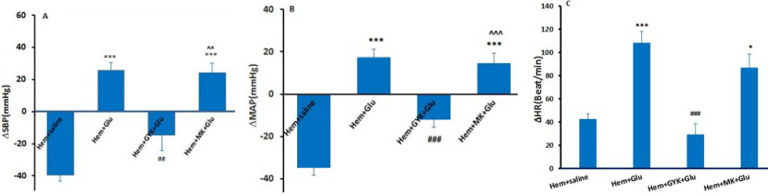
Maximum changes (∆) of systolic blood pressure (∆SBP, A), mean arterial pressure (∆MAP, B), and heart rate (∆HR, C) following saline, L- glutamate, GYK+ L-Glu, and MK-801+ L-Glu microinjection in the lPAG column in Hem hypotensive rats

In this experiment, Hem hypotension was induced at first, then L-Glu alone and together with GYK and MK-801 were microinjected into the lPAG. After microinjection of L-Glu, a significant increase in ΔSBP (25.55± 5.1 mmHg, *P*<0.05), ΔMAP (17.2± 4.2 mmHg; *P*<0.001), and ∆HR (108.4±9.4 Beat/min; *P*<0.001) was observed in comparison with the Hem+ saline (Figure 4). The changes of ΔSBP, ΔMAP, and ∆HR also were significant vs the saline group (*P*<0.01-*P*<0.001). 

During Hem, receiving the GYK before of L-Glu (Hem+ GYK+L-Glu group) could significantly attenuate ΔSBP (-14.72 ± 9.7 mmHg, *P*<0.01), ΔMAP (-12.2± 3.5 mmHg; *P*<0.001), and ∆HR (29.37±9.2 Beat/min; *P*<0.001) induced by the L-Glu group. However, GYK could not completely reverse the effect of L-Glu, so ΔSBP and ΔMAP were significantly more than the Hem (*P*<0.05 to *P*<0.01; Figure 4). In another group, after inducing Hem, MK-801 (an NMDA receptor antagonist) before L-Glu was microinjected into the lPAG (Hem+MK-801+L-Glu group). The results showed that MK-801 did not significantly attenuate ΔSBP (24.1 ± 5.9 mmHg) and ΔMAP (14.52 ± 4.8 mmHg) compared to the Hem+ -Glu group. So, these parameters in Hem+MK-801+L-Glu group were significantly higher than in Hem+GYK+L-Glu (*P*<0.01 to *P*<0.001; Figure 4).

## Discussion

Our findings revealed that the L-Glu injection within the lPAG raised the cardiac and vascular parameters in normal conditions. Also, microinjection of the NMDA (GYK) and non-NMDA (MK-801) antagonist alone and co-injection with L-Glu revealed that the cardiovascular impact of the non-NMDA receptors of lPAG is more significant than the NMDA receptors. In Hem hypotensive condition, microinjection of GYK and MK-801 alone could not significantly alter cardiac and vascular variables. In contrast, L-Glu alone and co-injection with MK-801 increased hypotensive responses, while co-injection of L-Glu with GYK did not improve the hypotensive effect. 

One of the known effects of lPAG is its role in defensive reactions. The defensive reactions are associated with active coping strategies (such as freezing, increased cardiovascular responses, and contraction of visceral vessels) and inactive coping strategies (such as immobility and sympathetic inhibition) (25). It is noted that the lPAG is involved in active coping (26). Therefore, its response to life-threatening factors is an increase in cardiovascular and respiratory parameters (26, 27). The exact excitatory cardiovascular effect of this area is currently unknown but may be mediated by neurons that present in lPAG or neural projections to this area. Previous research has revealed that glutamate has excitatory impacts on the cardiovascular network (26, 28). Furthermore, it has been shown that injection of D,L-homocysteic acid (DLH) into the lPAG increases blood pressure and HR (26). In line with the mentioned data, in our study, microinjection of L-Glu within the lPAG of rats with normal BP also caused raised arterial blood pressure and HR, which confirmed the excitatory role of L-Glu in the cardiovascular function of lPAG. Due to the existence of non-NMDA and NMDA receptors in the lPAG, to determine which glutamate receptor was involved in cardiovascular effects, MK-801 and GYK, the antagonists of NMDA and non-NMDA receptors, were separately microinjected into lPAG. GYK and MK-801 alone could not affect HR and blood pressure significantly. The lack of cardiovascular effects of antagonists may be due to low glutamate secretion under anesthesia conditions. Therefore, L-Glu was co-injected with MK-801 and GYK in separate groups to prove the role of receptors. It was observed that GYK could significantly attenuate the pressor outcome induced by L-Glu. Meanwhile, MK-801 had no significant effect. Thus, we suggested that the circulatory impact of L-Glu in the lPAG is predominantly facilitated by non-NMDA receptors. Similar to our experiment, the involvement of glutamate receptors in cardiovascular control has also been pointed out in former studies. For example, Zhang *et al*. show the impact of each NMDA and non-NMDA receptor of NTS in cardiovascular and baroreflex regulation (26). Also, Busnardo *et al.* reported that the non-NMDA and NMDA receptors of PVN are engaged in cardiovascular responsiveness in Hem (29). In addition, it has been indicated that the role of NMDA in central cardiovascular effect is higher than non-M NMDA (25, 30). VlPAG, for example, shows that the NMDA receptor of glutamate is more effective than the NMDA receptor (31). However, in this study, the function of the non-NMDA receptor in the lPAG is greater than the NMDA receptor. The mechanism(s) of this effect of non-NMDA in this area has not been determined and needs to be evaluated.

 The lPAG is one of the important regions in physical stressors (5), and studies have shown that this region can effectively control physical stress through projections to the NTS and RVLM (5, 6). It has been identified that this region also receives sensory inputs from the spinal cord (32). These inputs are one of the stimulating factors for physical stress, such as hemorrhage (5, 32). Based on this evidence, we assessed the possible contribution of the glutamatergic system of lPAG to Hem’s hypotensive condition. Microinjection of GYK and MK-801 by itself into this area in Hem hypotensive rats could not significantly alter cardiovascular parameters. In contrast, microinjection of L-Glu in this condition could improve hypotension and increase HR. Additionally, microinjection of GYK and MK-801 before L-Glu in this condition indicates that the effect of L-Glu was significantly attenuated only by GYK. Based on this effect, it is suggested that the effect of L-Glu in the Hem condition is similar to the basic condition and mediated by non-NMDA receptors. Our results show that microinjection antagonists of non-NMDA and NMDA receptors in Hem hypotensive situations did not change cardiovascular parameters. Therefore, we suggest that the endogenous glutamatergic system of lPAG does not have the power to improve hypotension caused by hemorrhage. In addition, L-Glu injection could improve Hem hypotensive condition and increased arterial pressure and heartbeat. The reason for this effect is unclear and requires further studies, but we speculate that the concentration of injected L-Glu was higher than the endogenous glutamate. Therefore, exogenous glutamate could increase cardiovascular parameters.

 The neural pathway through which the glutamatergic system of the lPAG exerts its cardiovascular effects is not well defined. However, It has been shown that there are projections from lPAG to the RVLM (an important sympathoexcitatory area) and IML (8). Therefore, we speculate that this pathway (lPAG-RVLM – IML) may have a position in cardiovascular reactions of glutamate in the lPAG and, via stimulating the sympathetic system, lead to increased cardiovascular parameters (33). Projections from lPAG to higher brain centers such as the hypothalamus have also been reported (34, 35), and it is possible that glutamate, through its effect on the hypothalamus, increases the secretion of vasopressin and consequently increases cardiovascular responses. The connection of lPAG with the NTS has also been identified (36). In addition, both non-NMDA and NMDA receptors of the NTS are engaged in the baroreceptor’s function. Therefore, it is possible that during Hem hypotensive condition, baroreceptors are stimulated, and via non-NMDA receptors of both lPAG and NTS areas adjust blood pressure and HR.

Comparing the effect of GYK under normal and Hem conditions showed that the hypotensive effect of GYK did not differ between the two conditions. Additionally, microinjection of MK-801 alone and co-injection with glutamate had no significant effect on hypotension induced by Hem, indicating that NMDA receptors in this region are not associated with its cardiovascular responses. 

 Based on baroreflex function, we expect HR to decrease as blood pressure increases. However, in this experiment, both parameters are increased, which shows that the baroreflex is weakened in the presence of L-Glu. The mechanisms of this impact are unclear; however, the pathways regulating HR and blood pressure in lPAG may differ. As lPAG has projections to both the RVLM(37) and NTS(36), we hypothesize that lPAG projection to NTS plays a role in controlling HR, and those projections to RVLM are involved in blood pressure control. However, future research is necessary to validate this opinion.

**Table 1:** Cardiovascular responses resulting from saline microinjection into the lateral periaqueductal gray (lPAG) in anesthetized rats

**Parameters**	**MAP (mmHg)**	**SBP (mmHg)**	**HR (Beat/min)**
Pre injection	112±11.15	134.9±9.4	380.9±20.12
Post injection	113.4 ± 12.6	136.3 ± 11.2	383.3 ± 17.6

Data was analyzed using an independent t-test and is shown as mean ± SEM. n = 6.

HR: Heart rate; MAP: Mean arterial pressure; SBP: Systolic blood pressure

The data is expressed as mean ± SEM, n = 6. One-way ANOVA and *post hoc *Tukey's test were used to analyze the changes. *, *P*<0.05 and**, *P*<0.01 compared to the Saline group.

GYK: 1-(4-aminophenyl)-4-methyl-7,8-methylenedioxy-5H-2,3-benzodiazepine , lPAG : Lateral periaqueductal gray matter

Data are expressed as mean ± SEM. n = 6. One-way ANOVA and *post hoc *Tukey's test were used to analyze the changes. ***P<0.001, **P<0.01, *P<0.05 compared to the Saline group

## *P*<0.01 compared to the L-Glu group. GYK: 1-(4-aminophenyl)-4-methyl-7,8-methylenedioxy-5H-2,3-benzodiazepine , lPAG : Lateral periaqueductal gray matter , L-Glu; L-Glutamate

Data are expressed as mean ± SEM. n = 6. One-way ANOVA and *post hoc* Tukey's test were used to analyze the changes. ***P<0.001 compared to the saline group

GYK: 1-(4-aminophenyl)-4-methyl-7,8-methylenedioxy-5H-2,3-benzodiazepine , lPAG : Lateral periaqueductal gray matter , Hem:hemorrhage

Data are expressed as mean ± SEM. n = 6. One-way ANOVA and* post hoc* Tukey's test were used to analyze the changes. ***P<0.001, *P<0.05 compared to the control group. ## *P*<0.01 compared to the Hem group. ^^^ *P*<0.001 compared to the Hem+ L-Glu group.

GYK: 1-(4-aminophenyl)-4-methyl-7,8-methylenedioxy-5H-2,3-benzodiazepine , lPAG : Lateral periaqueductal gray matter, Hem:hemorrhage Glu:Glutamate

## Conclusion

The outcomes of the current experiment provided document for the excitatory cardiovascular role of the glutamatergic system of lPAG in normotensive conditions mediated by non-NMDA receptors. In hem-hypotensive situations, endogenous glutamate of lPAG does not have the power to improve hypotension caused by hemorrhage. In contrast, exogenous glutamate was able to elicit cardiovascular responses in hem-hypotensive animals.

## Declaration

No artificial intelligence tools were used in the writing of this manuscript.
